# Supplementary material for: ADAM17—A Potential Blood-Based Biomarker for Detection of Early-Stage Ovarian Cancer
Source: Cancers (Basel). 2021 Nov 6;13(21):5563. doi: 10.3390/cancers13215563 (PMC8583642; doi:10.3390/cancers13215563)
Supplement: Supplementary file 1 [file cancers-13-05563-s001.zip › cancers-1434202-supplementary.pdf]

cancers

Supplementary Materials: ADAM17—A Potential Blood-Based Biomarker for Detection of Early-Stage Ovarian Cancer

Christoph Rogmans, Jan Dominik Kuhlmann, Gerrit Hugendieck, Theresa Link, Norbert Arnold, Jörg Paul Weimer, Inken Flörkemeier, Anna-Christina Rambow, Wolfgang Lieb, Nicolai Maass, Dirk O. Bauerschlag and Nina Hedemann

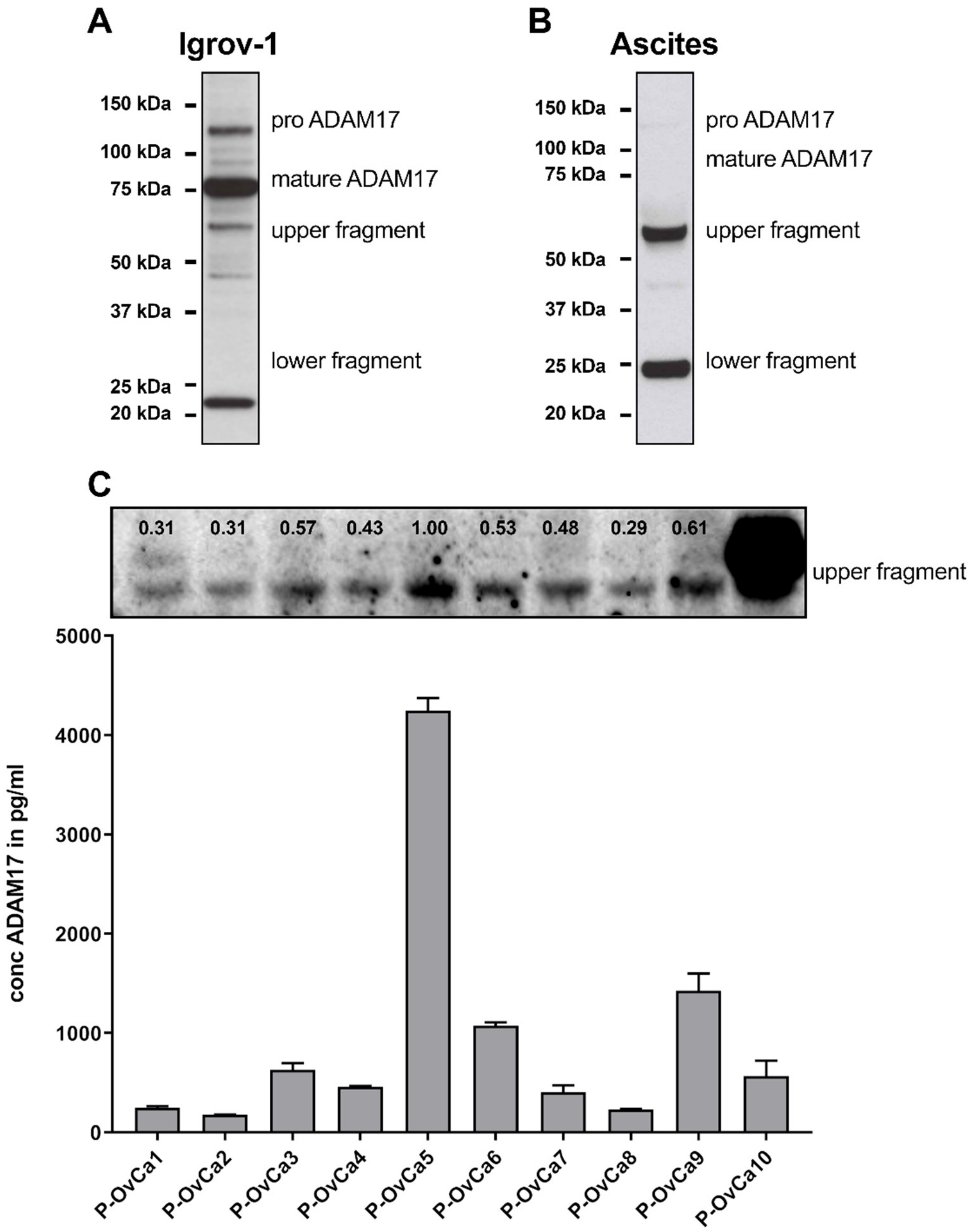

Figure S1. Validation of ADAM17 protein in lysates and ascites. Lysates of ovarian cancer cells (Igrov-1) (**A**) and ascites of an ovarian cancer patient (**B**) were analyzed by western blot to validate ADAM17 protein expression and identify the most prominent ADAM17 forms. In cell lysates four major bands were detected: 1. the proform at 120 kilo Dalton (kDa) containing the inhibitory prodomain, 2. the mature form 90 kDa, lacking the prodomain, 3. an upper fragment (55 kDa–60 kDa), being described as a secondary cleavage product of ADAM17 by other proteases like matrix-metallo proteases (MMPs) [31,32], and 4. a lower fragment 20-25 kDa fragment being described as a

membrane associated cleavage product [32]. In ascites samples, the upper and the lower fragment were most prominent. A slight band at 120 kDa was detected in addition. As presence and activity of MMPs in ascites fluid was shown by several studies, the upper fragment was further analyzed and quantified, to test if band intensities would correlate with ADAM17 values measured by ELISA [33, 34] (**C**). Band intensities were quantified by densitometry and relative levels displayed above the respective bands. The strongest band intensity was set to one. Except for sample 10, which was haemolysed and thus showed a differential band pattern, all relative levels of band intensities correlate with ADAM17 levels measured by ELISA (bargraph underneath and figure 2) of identical ascites samples 1–9.

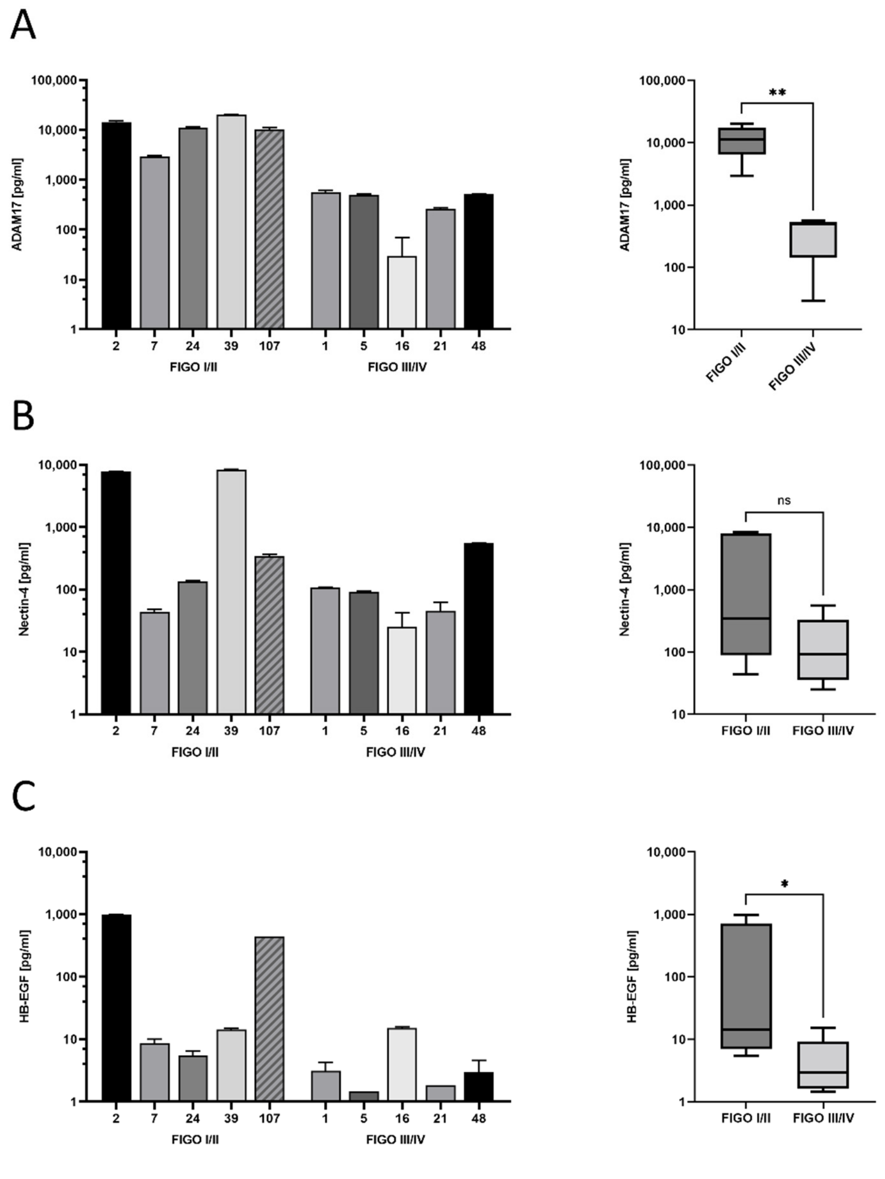

Figure S2. Protein levels of ADAM17 and its substrates in early and advanced FIGO stage. ADAM17 (**A**), Nectin-4 (**B**) and HB-EGF (**C**) concentrations were measured by ELISA and are displayed as technical duplicates + SD. Individual patient data are displayed on the left and the summarized FIGO groups of the identical data on the right as box plots. 10 patient samples were selected to compare early (patient number: 2, 7, 24, 39) to late (patient number: 1, 5, 16, 21, 48) FIGO stages. Additionally, one borderline patient (107) was included. Tendencies of ADAM17 and Nectin-4 inter-individually correlated and showed higher levels in early (mean: 342.7) compared to late stages (mean: 92.50) (Mann Whitney test, $p = 0.1111$). HB-EGF levels followed mostly similar trends and differed significantly between FIGO stages (mean: FIGO I/II: 14.26 vs. mean: FIGOIII/IV late 2.974)

(Mann Whitney test, $p = 0.0278$). Stars indicate significant differences: * $p < 0.05$, ** $p < 0.01$, ns: non-significant.

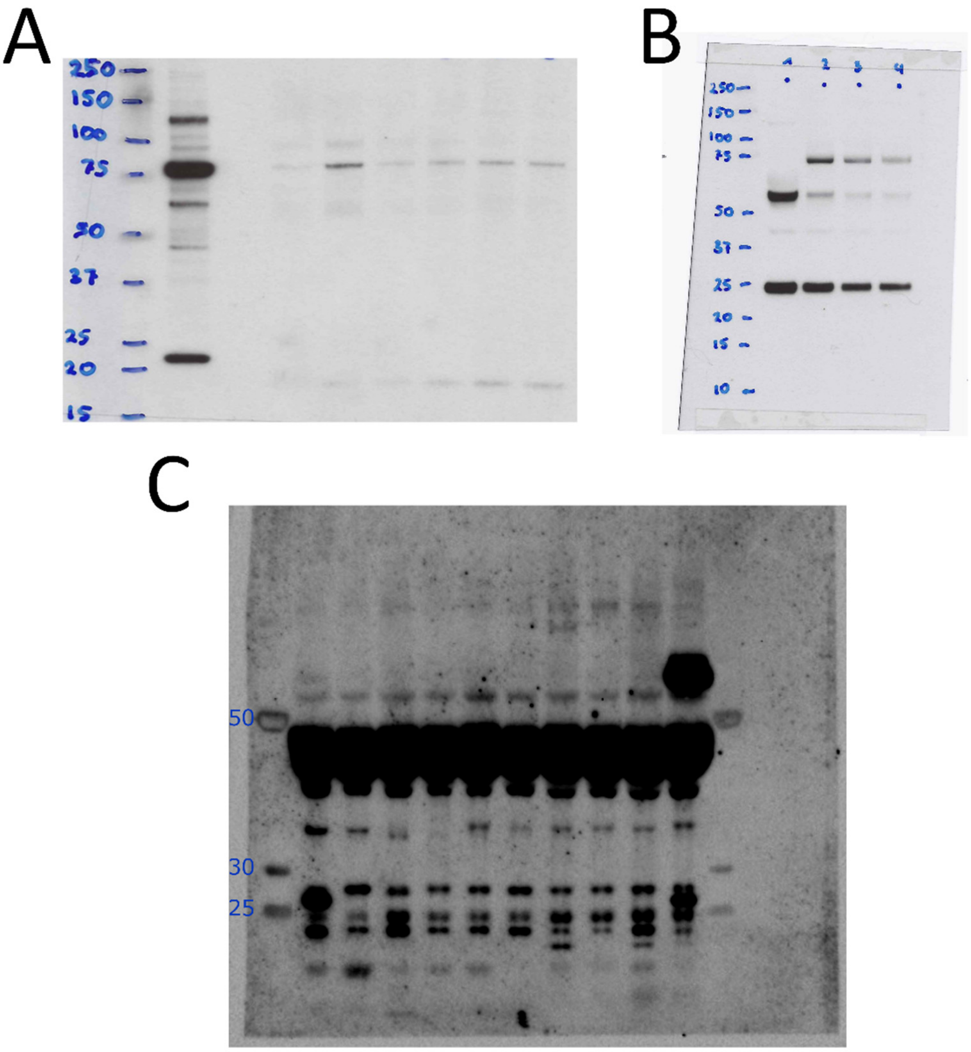

Figure S3. Uncropped western blots: Validation of ADAM17 protein in lysates and ascites. Lysates of ovarian cancer cells (Igrov-1) first lane (**A**) and ascites of an ovarian cancer patient first lane (**B**) were analyzed by western blot to validate ADAM17 protein expression and identify the most prominent ADAM17 forms. In cell lysates four major bands were detected: 1. the proform at 120 kilo Dalton (kDa) containing the inhibitory prodomain, 2. the mature form 90 kDa, lacking the prodomain, 3. an upper fragment (55 kDa–60 kDa), being described as a secondary cleavage product of ADAM17 by other proteases like matrix-metallo proteases (MMPs) [31,32], and 4. a lower fragment 20–25 kDa fragment being described as a membrane associated cleavage product [32]. In ascites samples, the upper and the lower fragment were most prominent. A slight band at 120 kDa was detected in addition. As presence and activity of MMPs in ascites fluid was shown by several studies, the upper fragment was further analyzed and quantified, to test if band intensities would correlate with ADAM17 values measured by ELISA [33,34] (**C**). Additional unspecific bands, were detected in the center of the blot (>25 kDa and <50 kDa). Except for sample 10, which was haemolyed and thus showed a differential band pattern, all relative levels of band intensities of the upper fragment (55 kDa–60 kDa) correlate with ADAM17 levels measured by ELISA (figure 2, figure S2C) of identical ascites samples 1–9.
